# Oncogenic *Kras* Initiates Leukemia in Hematopoietic Stem Cells

**DOI:** 10.1371/journal.pbio.1000059

**Published:** 2009-03-17

**Authors:** Amit J Sabnis, Laurene S Cheung, Monique Dail, Hio Chung Kang, Marianne Santaguida, Michelle L Hermiston, Emmanuelle Passegué, Kevin Shannon, Benjamin S Braun

**Affiliations:** 1 Department of Pediatrics, University of California San Francisco, San Francisco, California, United States of America; 2 The Eli and Edythe Broad Center for Regeneration Medicine and Stem Cell Research, University of California San Francisco, San Francisco, California, United States of America; University of Rochester Medical Center, United States of America

## Abstract

How oncogenes modulate the self-renewal properties of cancer-initiating cells is incompletely understood. Activating *KRAS* and *NRAS* mutations are among the most common oncogenic lesions detected in human cancer, and occur in myeloproliferative disorders (MPDs) and leukemias. We investigated the effects of expressing oncogenic *Kras^G12D^* from its endogenous locus on the proliferation and tumor-initiating properties of murine hematopoietic stem and progenitor cells. MPD could be initiated by *Kras^G12D^* expression in a highly restricted population enriched for hematopoietic stem cells (HSCs), but not in common myeloid progenitors. *Kras^G12D^* HSCs demonstrated a marked in vivo competitive advantage over wild-type cells. *Kras^G12D^* expression also increased the fraction of proliferating HSCs and reduced the overall size of this compartment. Transplanted *Kras^G12D^* HSCs efficiently initiated acute T-lineage leukemia/lymphoma, which was associated with secondary *Notch1* mutations in thymocytes. We conclude that MPD-initiating activity is restricted to the HSC compartment in *Kras^G12D^* mice, and that distinct self-renewing populations with cooperating mutations emerge during cancer progression.

## Author Summary

Ras proteins act as molecular switches that relay growth signals from outside the cell. This mechanism is often subverted in cancer, and Ras proteins are activated directly by *RAS* gene mutations in approximately one-third of human malignancies. We have modeled this in mice engineered to have a *Ras* mutation. These mice develop a disease similar to chronic leukemias in humans called myeloproliferative disorders. It is marked by a fatal accumulation of mature and immature cells in the blood and bone marrow. We investigated whether some or all of these neoplastic cells were immortal. In agreement with the “cancer stem cell” hypothesis, we found that immortal cells were extremely rare in the bone marrow of diseased mice. They were found only in the same cell populations that contain normal bone marrow stem cells. However, these cells had high rates of replication and produced large numbers of daughter cells. Furthermore, many mice went on to develop acute lymphoid leukemia after acquiring additional mutations in maturing lymphoid cells. These studies exemplify the evolution of malignant stem cells during cancer progression. They also highlight the importance of rare, long-lived cells in the genesis and, potentially, therapy of high-risk chronic leukemias caused by abnormal Ras proteins.

## Abstract 3

The*Kras^G12D^* oncogene causes excessive proliferation of stem cells, promoting their preferential expansion and initiating myeloproliferative disease.*Kras^G12D^* does not alter self-renewal, but secondary mutations in lymphoid progenitors allow progression to acute leukemia.

## Introduction

Self-renewal is integral to the malignant phenotype [1]. In principle, the ability of cancer cells to self-renew may be intrinsic to the compartment in which the tumor-initiating mutation occurs, or may be acquired as a consequence of mutations in more differentiated cells. The hematopoietic system has proven highly informative for addressing how cancer-associated mutations and cell of origin interact to establish malignant self-renewing populations. Accumulating evidence supports the idea that many hematopoietic malignancies exist in a hierarchy of differentiation with only a minor population capable of propagating and maintaining the disease in vivo [2]. These cells are termed leukemia-initiating cells or leukemia stem cells (LSCs), and manifest some biologic properties of normal hematopoietic stem cells (HSCs). However, the precise relationship between these populations is uncertain and appears to depend, in part, on both the leukemia subtype and on the effects of specific mutations. For example, overexpressing *MLL* fusion proteins found in human acute myeloid leukemia transforms both murine HSCs and more differentiated progenitors [3,4]. By contrast, inactivation of the *JunB* transcription factor must occur in the HSC compartment for initiation of myeloid malignancies [5]. These proof-of-concept experiments underscore the importance of understanding how oncogenes and tumor suppressors that are commonly mutated in human cancers perturb self-renewal and growth control. Importantly, the functional characteristics of LSCs that distinguish them from HSCs and how these properties are modulated by oncogenes are poorly understood.

*RAS* gene mutations are highly prevalent in pancreatic (>80%), colorectal (40%–50%), endometrial (40%), lung (30%), and cervical cancers (20%–30%), as well as in myeloid malignancies (20%–40%) [6]. Of the genes in the canonical *RAS* family, *KRAS* accounts for ∼90% of cancer-associated mutations, whereas *HRAS* mutations are rare. In hematologic cancers, *NRAS* is mutated 2–3 times more often than *KRAS* [6]. Cancer-associated *RAS* mutations, which introduce amino acid substitutions at codons 12, 13, or 61, result in oncogenic Ras proteins that accumulate in the active, GTP-bound conformation because of defective guanine nucleotide hydrolysis [7]. Elevated levels of GTP-bound Ras, in turn, deregulate signaling in cancer cells by altering the activation of effector cascades that include the Raf/MEK/ERK, phosphatidylinositol 3-kinase (PI3K)/Akt, and Ral-GDS pathways [8].

Chronic and juvenile myelomonocytic leukemias (CMML and JMML) are aggressive myeloid malignancies that are classified as myeloproliferative disorders (MPDs) [9]. Both diseases are characterized by leukocytosis with excess monocytes in blood and bone marrow, and by significant infiltration of malignant myeloid cells into the liver, spleen, and other organs. Hyperactive Ras is strongly implicated in the pathogenesis of JMML and CMML. Somatic *NRAS* and *KRAS* mutations are found in ∼40% of CMML specimens [10,11], and ∼85% of JMML patients have mutations in *KRAS, NRAS, NF1,* or *PTPN11* (reviewed in [12]). The latter two genes encode proteins that regulate Ras-GTP levels. Importantly, children with germline mutations in *NF1* or *PTPN11* are at markedly increased risk of developing JMML, which argues strongly that deregulated Ras signaling can initiate this MPD [12]. This hypothesis is further supported by studies of *Nf1, Kras*, and *Ptpn11* mutant mice, all of which develop MPDs that resemble JMML and CMML [13–16]. Mutations that alter other signaling molecules also cause MPD. For example, the *BCR-ABL* fusion gene is the hallmark of chronic myeloid leukemia, and *JAK2* mutations are found in nearly all cases of polycythemia vera (reviewed in [17]). While mutations affecting signaling molecules are found in nearly all types of MPD, additional cooperating mutations are rare. MPDs are therefore genetically straightforward and tractable malignancies for understanding how aberrant signal transduction contributes to cancer by perturbation of stem and progenitor cell fates.

Mice expressing oncogenic *Kras^G12D^* in hematopoietic cells develop a fatal MPD with 100% penetrance that is characterized by leukocytosis, splenomegaly, and anemia, with death at approximately 3 mo of age [13,14]. In this system, injecting *Mx1-Cre, Kras^LSL-G12D^* mice with polyinosinic-polycytidylic acid (pIpC) induces expression of Cre recombinase, which removes an inhibitory loxP-STOP-loxP (LSL) element and activates the *Kras^G12D^* allele (we hereafter refer to *Mx1-Cre, Kras^LSL-G12D^* mice that have been treated with pIpC as *Kras^G12D^* mice). *Mx1-Cre, Kras^LSL-G12D^* mice provide a robust experimental system for investigating how expressing oncogenic *Kras* from the endogenous promoter affects HSCs and their progeny in vivo. Here, we identify tumor initiating cells in the *Kras^G12D^* model of MPD and find that, unlike some myeloid oncogenes, *Kras^G12D^* does not confer aberrant self-renewal properties to committed progenitor cells. However, small numbers of primitive *Kras^G12D^* cells can initiate MPD. This population shows excessive proliferation and rapidly dominates multilineage hematopoiesis in vivo. These data indicate that hyperactive Ras signaling is sufficient for the competitive advantage demonstrated by mutant HSCs and further implicate the HSC compartment as critical for therapy of JMML and CMML. Transplanted *Kras^G12D^* HSCs efficiently initiate T lineage acute lymphoblastic leukemia/lymphoma (T-ALL), which is associated with *Notch1* mutations and with acquisition of LSC activity in differentiating thymocytes. These results further demonstrate that distinct self-renewing populations can arise through cooperating oncogenic mutations during cancer progression.

## Results

### A Small *Kras^G12D^* Population Dominates Both Primitive and Differentiated Hematopoietic Compartments in *Mx1-Cre, Kras^LSL-G12D^* Mice

**Figure 1:**
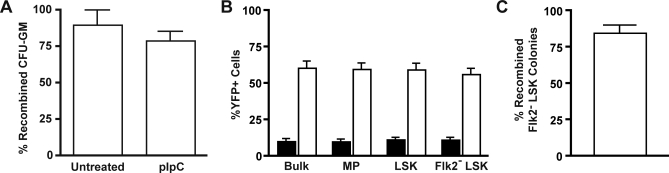
Spontaneous *Kras^G12D^* Activation in CFU-GM and HSCs from *Mx1-Cre, Kras^LSL-G12D^* Mice (A) Bone marrow was harvested at 24 d or 5 wk of life from mice that either received a single dose of pIpC at 21 d of life or were left untreated, and plated in methylcellulose medium with GM-CSF. Individual colonies were isolated and genotyped by PCR to assess for Cre-mediated excision of the LSL cassette (*n =* 18 colonies from two uninjected animals, and 34 colonies from two injected animals, compiled from two independent experiments; error bars show SEM). (B) *Mx1-Cre* (filled bars) and *Mx1-Cre, Kras^LSL-G12D^* (open bars) mice that inherited an *LSL-YFP* reporter were sacrificed at 5 wk of life, without ever being injected with pIpC. Expression of YFP requires excision of the *LSL* cassette from the reporter gene by Cre recombinase. The percentage of YFP^+^ cells in all bone marrow cells (Bulk), Lin^−^ c-kit^+^ Sca1^−^ myeloid progenitors (MP), LSK cells, and Flk2^−^ LSK cells was assessed by flow cytometry. (*n =* 4 for WT and for *Mx1-Cre, Kras^LSL-G12D^* mice compiled from three independent experiments; error bars show SEM). (C) Flk2^−^ LSK cells were sorted from bone marrow of 4-wk-old *Mx1-Cre, Kras^LSL-G12D^* mice that had not received pIpC, and plated in methylcellulose medium for a culture period of 14 d. Individual colonies were genotyped by PCR (*n =* 44 colonies from 6 mice in 3 independent experiments; error bar shows SEM).

Although basal *Mx1-Cre* activity is low [18,19], many *Mx1-Cre, Kras^LSL-G12D^* mice that are not injected with pIpC ultimately succumb with MPD ([14] and unpublished data). This observation suggests that HSCs and/or progenitor cells that activate *Kras^G12D^* expression have a substantial proliferative advantage in vivo. To assess the kinetics of this process, we analyzed recombination in the myeloid progenitors of young *Mx1-Cre, Kras^LSL-G12D^* mice that were not treated with pIpC. Bone marrow cells from 3–5-wk-old animals were plated in methylcellulose medium to enumerate granulocyte-macrophage colony forming unit progenitors (CFU-GM), and individual myeloid colonies were genotyped by PCR. Surprisingly, nearly all CFU-GM in untreated animals were recombined as early as 3 wk of age (Figure 1A). We then examined more primitive populations in *Mx1-Cre, Kras^LSL-G12D^* mice crossed to a *ROSA26* yellow fluorescent protein (YFP) reporter strain (Figure 1B) [20]. In these mice, cells expressing Cre are identified by YFP expression. The frequency of YFP+ cells in wild-type (WT) mice was within the expected range of background Cre expression [18,19]. However, we found a much higher incidence of YFP expression in bone marrow cells of *Mx1-Cre, Kras^LSL-G12D^, ROSA26-YFP* mice. This result was consistent among all populations analyzed, including the primitive Flk2^−^ Lin^−/lo^ Sca1^+^ c-kit^+^ (Flk2^−^ LSK) compartment, which is highly enriched for HSCs [21]. To confirm that YFP expression correlated with *Kras^G12D^* expression, we also directly genotyped colonies formed by single Flk2^−^ LSK cells. This method again revealed a predominance of *Kras^G12D^*-expressing cells in naïve *Mx1-Cre, Kras^LSL-G12D^* mice (Figure 1C). Together, these data are consistent with an advantage for Cre-expressing cells in untreated *Mx1-Cre, Kras^LSL-G12D^* mice.

### Increased Cell Cycle Entry of *Kras^G12D^* HSCs

**Figure 2:**
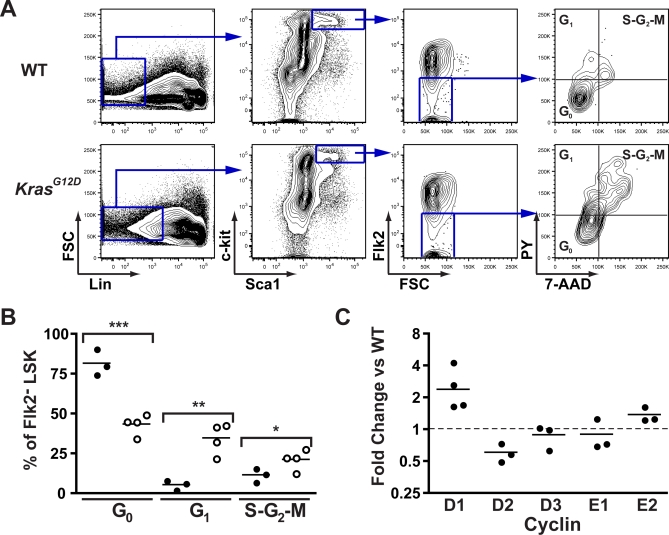
Increased Proliferation of *Kras^G12D^* HSC Bone marrow from 5-wk-old WT and *Kras^G12D^* mice was stained with 7-AAD and pyronin Y (PY) for DNA and RNA quantitation, along with surface markers for Flk2^−^ LSK cells. (A) Gating is shown for cell cycle analysis of WT and *Kras^G12D^* Flk2^−^ LSK cells. (B) Summary of replicate samples from WT (closed circles) and *Kras^G12D^* (open circles) mice (*n =* 3 or 4 as shown; compiled from two independent experiments). Means and SEM are WT: G_0_ 81.8 ± 4.72, G_1_ 5.66 ± 1.76, S-G_2_-M 11.7 ± 2.56; and *Kras^G12D^*: G_0_ 43.6 ± 3.19, G_1_ 34.9 ± 4.85, S-G_2_-M 21.4 ± 3.14. *p*-Values by unpaired *t*-test are indicated: ***, *p <* 0.001; **, *p <* 0.01; *, *p <* 0.05. (C) RNA from doubly sorted Flk2^−^ LSK cells was isolated, and quantitative PCR performed on cDNA to test expression levels of selected cyclins (*n =* 3 or 4 as shown; bar shows geometric mean). Results are expressed as fold change in expression compared to WT Flk2^−^ LSK cells, after normalization to β-actin expression. These results represent three or four independent experiments as shown, each performed with pooled bone marrow from three to five animals. The geometric mean of cyclin D1 expression is 2.4-fold over WT (95% confidence interval 1.2–4.9). Purity of sorted cells is shown in Figure S1.

The apparent outgrowth of *Kras^G12D^* Flk2^−^ LSK cells in mice that were not injected with pIpC suggested that *Kras^G12D^* expression might increase proliferation in this compartment. To test this hypothesis, we stained bone marrow cells collected from *Kras^G12D^* mice and WT littermates 2 wk after pIpC treatment with antibodies to cell surface proteins and with dyes that stain DNA and RNA (7-aminoactinomycin D [7-AAD] and pyronin Y, respectively). These studies revealed a significant reduction in the number of quiescent Flk2^−^ LSK cells in *Kras^G12D^* animals, which are identified by having a 2n DNA content and low pyronin Y staining (Figure 2A and 2B). Whereas the Flk2^−^ subset of WT LSK contains roughly 80% cells in the G_0_ phase of the cell cycle, *Kras^G12D^* Flk2^−^ LSK are only 50% quiescent. As an initial exploration of mechanisms regulating cell cycle progression, we analyzed D- and E-type cyclin expression by quantitative PCR in sorted Flk2^−^ LSK cells from *Kras^G12D^* and WT mice. *Kras^G12D^* Flk2^−^ LSK cells had significantly higher expression of cyclin D1 (Figure 2C).

### Self-Renewal Is Restricted to Primitive Hematopoietic Cells in *Kras^G12D^* Mice

**Figure 3:**
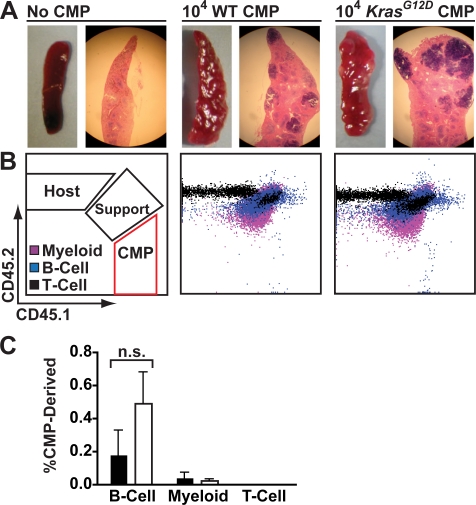
*Kras^G12D^* Does Not Lead to CMP Self-Renewal Sorted CD45.1 CMPs were transplanted into lethally irradiated CD45.2 recipients. (A) Spleens were harvested for CFU-S_8_ colony analysis 8 d after transplantation of 10^4^ *Kras^G12D^* or WT CMPs. Gross images and hematoxylin/eosin stained sections are shown. (B) Peripheral blood of animals analyzed 1 mo after transplantation of 10^4^ *Kras^G12D^* or WT CMPs along with 10^6^ CD45.1/CD45.2 heterozygous nucleated bone marrow cells for radioprotection (Support). Costaining with surface markers allowed detection of contributions to myeloid (purple), B (blue), and T (black) lineages. (C) Percentages of CMP-derived progeny within the indicated lineages found in peripheral blood 1 mo after transplantation of WT (filled bars) or *Kras^G12D^* (open bars) CMPs (*n =* 4 in two independent sorts).

**Figure 4:**
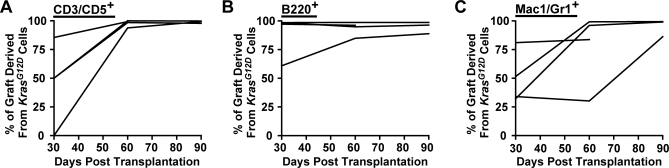
Transplanted *Kras^G12D^* Flk2^−^ LSK Cells Outperform WT LSK Cells in Competitive Reconstitution Lethally irradiated recipients (*n =* 4 in a single experiment) were transplanted with 500 each of *Kras^G12D^* and WT Flk2^−^ LSK cells, then bled monthly until sacrifice. Graphs show the proportion of graft-derived cells that came from the *Kras^G12D^* donor in circulating (A) T cell (CD3/CD5+), (B) B cell (B220+), and (C) myeloid (Mac1/Gr1+) populations. This was calculated by (1) gating for graft-derived cells (CD45.1 and CD45.1/CD45.2) within a lineage, and then (2) determining the fraction of *Kras^G12D^*-derived cells (CD45.1) within this subset.

Bone marrow mononuclear cells from *Kras^G12D^* mice display a hypersensitive pattern of CFU-GM progenitor growth, which is a cellular hallmark of JMML [12–14]. We found that this abnormal CFU-GM activity resides primarily in the common myeloid progenitor (CMP) compartment, and that these cells demonstrate enhanced proliferation in vivo ([22] and unpublished data). To investigate if this population could initiate and maintain MPD, we collected Lin^−/lo^ Sca1^−^ c-kit^+^ CD34^+^ FcγRII/III^−^ CMPs from 5-wk-old *Kras^G12D^* mice and WT littermates by FACS, and transferred 10,000 of these cells into lethally irradiated recipients with 10^6^ WT marrow cells for radioprotective support. Transplanted tester cells, recipient cells, and support cells were marked by expression of different isoforms of CD45, allowing them to be distinguished by flow cytometry. Transplanted *Kras^G12D^* or WT CMPs demonstrated robust day 8 spleen colony-forming unit (CFU-S_8_) activity, with colony size somewhat larger for *Kras^G12D^* input CMPs (Figure 3A). However, we detected less than 0.1% of circulating myeloid cells derived from transplanted *Kras^G12D^* or WT CMPs 1 mo after transplantation (Figure 3B and 3C). As expected from previous studies [23,24], *Kras^G12D^* and WT CMPs made minor contributions to the circulating B cell compartment with a statistically insignificant trend towards greater B cell production from *Kras^G12D^* cells. Taken together, these data indicate that *Kras^G12D^* CMPs do not initiate a hematologic disease. By contrast, transferring 500 Flk2^−^ LSK cells from *Kras^G12D^* animals into lethally irradiated recipients rapidly resulted in durable multilineage reconstitution. *Kras^G12D^* cells dominated the T cell and B cell compartments sooner and to a higher degree than the progeny of WT Flk2^−^ LSK cells in control animals. The myeloid series demonstrated a more variable time course, but *Kras^G12D^* derived cells also eventually out-competed WT cells (Figure 4).

**Figure 5:**
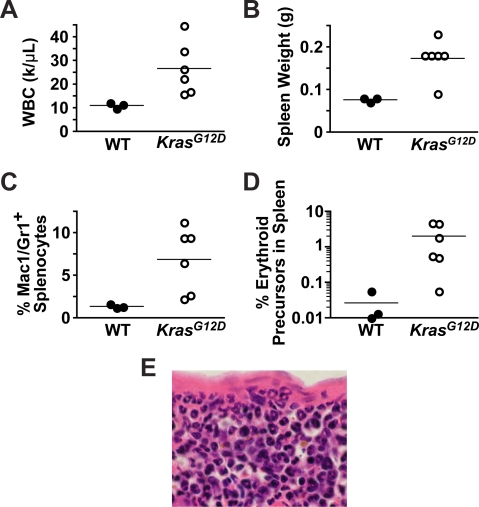
Transplanted *Kras^G12D^* Flk2^−^ LSK Cells Induce MPD Mice were analyzed 3 mo after transplantation with either *Kras^G12D^* or WT Flk2^−^ LSK cells for evidence of MPD (*n =* 3 WT, *n =* 6 *Kras^G12D^* in a single experiment). (A) Numbers of circulating leukocytes (WBC). (B) Spleen weights. (C) Percentages of myeloid cells (Mac1+ and/or Gr1+) in the spleen. (D) Percentages of erythroid precursors (nucleated and TER119+) in the spleen. (E) A representative splenic section demonstrating infiltration by mature myeloid cells (ring-shaped nuclei) and erythroblasts (small dense nuclei).

Recipients of *Kras^G12D^* Flk2^−^ LSK cells that were euthanized 3 mo after transplantation had mild to moderate MPD, manifested as leukocytosis and splenomegaly with myeloid and erythroid infiltration (Figure 5). As discussed below, we also found that all recipients of *Kras^G12D^* Flk2^−^ LSK cells developed T-ALL 2–4 mo after adoptive transfer. Although early mortality from T-ALL precluded analyzing recipient mice beyond 3 mo, *Kras^G12D^* Flk2^−^ LSK cells recapitulate the essential features of MPD seen in the original *Mx1-Cre, Kras^LSL-G12D^* model [13,14].

### HSCs Are Less Frequent in *Kras^G12D^* Mice but Have Enhanced Repopulating Ability

**Figure 6:**
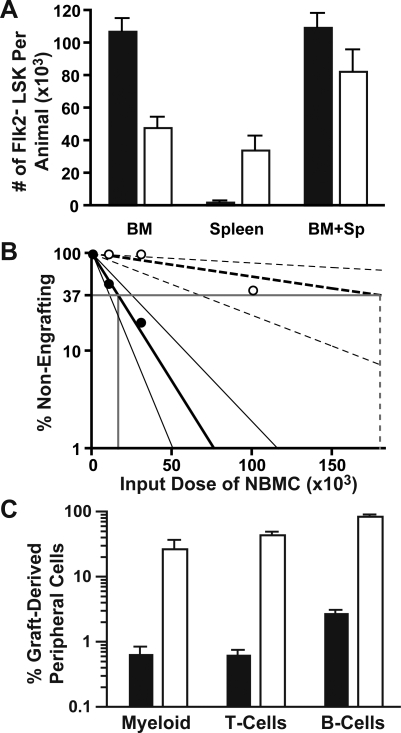
HSC Number and Proliferative Capacity in *Kras^G12D^* Mice HSCs were analyzed in *Kras^G12D^* mice or WT littermates that were treated with pIpC at 21 d of age and then sacrificed at 35 d of age. (A) Total numbers of Flk2^−^ LSK cells in *Kras^G12D^* mice (open bars) or WT mice (filled bars) were quantified by flow cytometry in bone marrow (*p <* 0.001, *t*-test) and spleen (*p <* 0.01); total numbers (spleen + marrow) are also shown (*p =* 0.12). *n =* 6 mice per genotype, and error bars show SEM; data are pooled from two independent experiments. Frequencies of Flk2^−^ LSK cells among viable nucleated cells were multiplied by nucleated bone marrow cell counts in femurs and tibias and then scaled to estimate total bone marrow numbers using published distributions [75]. (B) Whole bone marrow from *Kras^G12D^* (open circles) or WT littermates (filled circles) was tested for repopulating activity in a limit dilution transplantation assay. The calculated values for frequencies of repopulating units were 1 in 16,610 nucleated bone marrow cells (NMBC) for WT marrow and 1 in 180,404 for *Kras^G12D^* marrow. Narrow lines designate 95% confidence intervals (1:7,338 to 1:37,598 for WT and 1:68,953 to 1: 471,998 for *Kras^G12D^*; frequencies in WT versus *Kras^G12D^* marrow are significantly different with *p =* 0.0003 by two-tailed *t*-test). Outcomes of individual experiments are described in Table S1. (C) Mice that engrafted after transplantation with limiting numbers of whole bone marrow cells (1 × 10^4^ for WT, 1 × 10^5^ for *Kras^G12D^*) were bled at 2 mo to determine the percentage of circulating myeloid (Mac1/Gr1+), B-lineage (B220+), and T-lineage (CD3/CD5+) cells that were derived from the transplanted marrow. Filled bars represent mice receiving WT marrow (*n =* 2), open bars represent mice receiving *Kras^G12D^* marrow (*n =* 3; error bars show SEM; note logarithmic scale).

We next asked how *Kras^G12D^* expression affects HSC function. Immunophenotypic analysis revealed a 2-fold reduction in the number of marrow Flk2^−^ LSK cells in *Kras^G12D^* mice 2 wk after pIpC injection (Figure 6A). This was mostly offset by an increased number of splenic Flk2^−^ LSK cells. The reduction in marrow Flk2^−^ LSK cells persisted in older animals (Figure S2). We also performed limit dilution studies to assess functional HSC activity. In these experiments, lethally irradiated recipients received decreasing numbers of whole bone marrow cells from either *Kras^G12D^* mice or WT littermates that had been injected with pIpC 2 wk earlier. Recipients were bled monthly, and flow cytometry was performed to assess whether CD45.1^+^ donor cells were able to provide durable (>2 mo) multilineage (myeloid, B cell, and T cell) engraftment. These studies demonstrated a striking 10-fold decrease in the number of long-term repopulating stem cells in *Kras^G12D^* animals compared to the WT littermate controls (Figure 6B; Table S1).

Progression of MPD despite a reduction in the size of the HSC compartment suggests an increased production of mature cells by each *Kras^G12D^* HSC. To address this possibility, we examined the patterns of reconstitution from either *Kras^G12D^* or WT HSCs in mice that received the limit dilution dose (1 × 10^5^ *Kras^G12D^* and 1 × 10^4^ WT bone marrow cells). As ∼50% of these recipients were engrafted with donor cells, the Poisson distribution predicts that approximately 70% of the engrafting mice received a single HSC and ∼24% received two HSC. By 2 mo after transplantation, donor *Kras^G12D^* marrow cells made a markedly greater contribution to recipient hematopoiesis than WT cells (Figure 6C).

We were able to compare repopulation of the stem cell compartment by WT and *Kras^G12D^* HSCs in a few lethally irradiated recipient mice before the onset of T-ALL. In these experiments, lethally irradiated recipients (CD45.2) were reconstituted with equal numbers of *Kras^G12D^* (CD45.1) and WT (CD45.1/CD45.2) Flk2^−^ LSK cells, as well as 10^6^ CD45.2 whole bone marrow cells (CD45.2) for radioprotection. Whereas only one of six recipients euthanized 2 mo after transplantation showed a clear bias towards *Kras^G12D^*-derived Flk2^−^ LSK cells, mice that survived for 3 mo without evidence of diffuse T-ALL demonstrated an overwhelming bias towards *Kras^G12D^*-derived Flk2^−^ LSK cells, myeloid progenitors, and mature myeloid cells (Figure S3).

### T-ALL Arises from Transplanted *Kras^G12D^* HSCs in Thymocytes That Acquire Somatic *Notch1* Mutations

**Figure 7:**
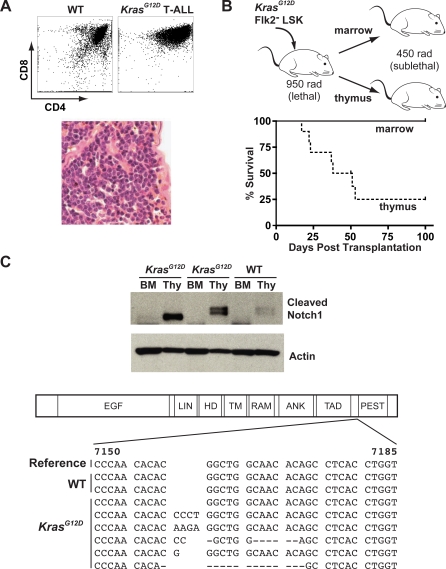
Transplanted *Kras^G12D^* Flk2^−^ LSK Cells Initiate T-ALLs That Contain *Notch1* Mutations (A) Flow cytometry of thymocytes harvested from moribund recipients of *Kras^G12D^* Flk2^−^ LSK cells shows an abnormal accumulation of CD4/CD8 double positive and immature CD8 single positive cells. Spleen histology demonstrates infiltration by monomorphic cells with open chromatin (hematoxylin/eosin). (B) Primary recipients (*n =* 5) received 500 *Kras^G12D^* Flk2^−^ LSK cells with or without an equal number of WT Flk2^−^ LSK cells after lethal irradiation (950 rad). These primary recipients were euthanized 2–3 mo later, and 10^6^ bone marrow cells or thymocytes were transferred into sublethally irradiated (450 rad) secondary recipients (two per primary mouse). Sublethal irradiation selectively permits transfer of acute leukemia but not *Kras^G12D^* HSCs or MPD [13]. Thymocytes, but not bone marrow cells, transferred T-ALL. (C) Cell lysates from thymocytes and bone marrow of animals euthanized 3 mo after transplantation with *Kras^G12D^* or WT HSCs were blotted with an antibody specific for cleaved Notch1. Three independent primary recipients are shown. Sequence analysis demonstrates frameshift mutations near the PEST domain of *Notch1* in thymocytes from five of six mice that received *Kras^G12D^* HSC, but not in recipients receiving WT HSCs alone (reference sequence from GenBank [http://www.ncbi.nlm.nih.gov/Genbank] accession number NM_008714).

Recipients that were injected with *Kras^G12D^* Flk2^−^ LSK cells, either alone or in a 1:1 ratio with WT Flk2^−^ LSK cells, uniformly became moribund 8–14 wk after transplantation. Examination of euthanized mice revealed massive thymic enlargement with an arrest in T cell development at the CD4/CD8 double positive stage, and variable infiltration of blast cells within the liver, spleen, and bone marrow (Figure 7A). Importantly, two of three animals from the bone marrow limit dilution transplantation assay that were engrafted with a single *Kras^G12D^* repopulating unit developed T-ALL that was identical to that seen in animals repopulated with 500 *Kras^G12D^* Flk2^−^ LSK cells (unpublished data).

Next, we asked if T-ALL arose within the bone marrow or the thymus of mice transplanted with *Kras^G12D^* HSCs. To do this, we took advantage of our prior observation that recipients conditioned with sublethal irradiation fail to engraft with *Kras^G12D^* bone marrow [13]. Therefore, sublethally irradiated recipients can exclusively select for hematologic malignancies with more aggressive biologic behavior. We isolated bone marrow cells and thymocytes from primary recipients of *Kras^G12D^* Flk2^−^ LSK cells that developed T-ALL, and injected each population into sublethally irradiated secondary recipients (Figure 7B). As expected, no animals demonstrated multilineage engraftment or MPD. Animals transplanted with thymocytes quickly succumbed with an identical T-ALL as primary recipients; however, none of the mice transplanted with bone marrow developed leukemia. Thus, whereas bone marrow-derived *Kras^G12D^* HSCs efficiently give rise to T-ALL, the T-ALL LSC population is initially restricted to the thymus in primary recipient mice.

These results suggested that one or more secondary mutations might have developed in a novel T-lymphoid clone. Somatic *NOTCH1* mutations are common in human and murine T-ALL [25–28]. To determine if a similar mechanism might contribute to the evolution of *Kras^G12D^* HSCs to T-ALL LSCs, we performed Western blot analysis to detect cleaved (activated) Notch1 protein. Cleaved Notch1 was observed in thymocytes from diseased primary recipients, but not in bone marrow cells (Figure 7C), a finding that is consistent with the secondary transplant data. Direct sequencing around the PEST domain uncovered frameshift mutations in exon 34 of *Notch1* in thymocytes from five of six animals transplanted with *Kras^G12D^* Flk2^−^ LSK cells that developed T-ALL, but no mutations in thymocytes from control mice that received WT HSCs alone and remained well. The presence of a somatically acquired *Notch1* mutation in a large fraction of the tumor provides a molecular indication of clonality.

### Thymic Abnormalities in *Kras^G12D^* Mice

**Figure 8:**
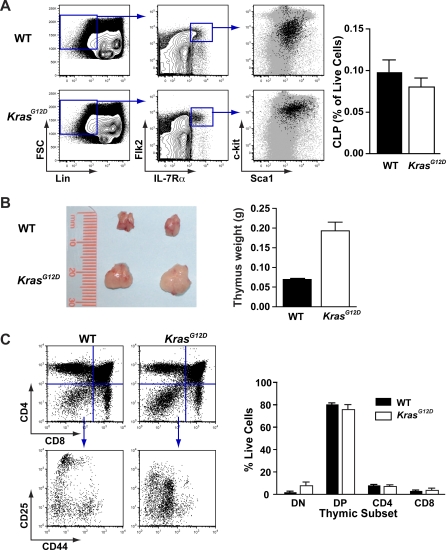
Abnormal T-lymphoid Development in *Kras^G12D^* Mice (A) Enumeration of Lin^−^ Flk2^+^ IL-7Rα^+^ c-kit^int^ Sca1^int^ common lymphoid progenitors (CLPs) in bone marrow of WT and *Kras^G12D^* mice. A typical comparison and gating strategy is shown, with the far right panel showing expression of c-kit and Sca1 in CLPs (black dots) compared to the larger population of Lin^−^ cells (gray dots). CLP frequencies are graphed (*n =* 5 WT and 6 *Kras^G12D^*; error bars show SEM and difference is not statistically significant by unpaired *t*-test). (B) Enlarged thymi in 7-wk-old *Kras^G12D^* mice compared with WT littermates; photograph shows a typical example, and graph shows data from a representative cohort (*n =* 3 mice and error bars show standard deviation; *p <* 0.05). (C) Flow cytometry of primary thymocytes for expression of CD4 and CD8, and also CD25 and CD44 expression within DN (CD4^−^ CD8^−^) cells; figure shows a representative example. Myeloid cells were excluded using Mac1 and Gr1 staining. Graph represents the frequency of thymocytes within in the live gate in DN, double positive (CD4+D8+, DP), and CD4 or CD8 single positive cells (*n =* 3 mice per genotype and error bars show SEM; *p* > 0.05 for all populations. Data are representative of three independent experiments).

The propensity of *Kras^G12D^* HSCs to generate T-ALL led us to investigate the effects of *Kras^G12D^* expression in early T-lineage cells. Flow cytometry of the bone marrow revealed no expansion of Lin^−^ Flk2^+^ IL-7Rα^+^ c-kit^int^ Sca1^int^ common lymphoid progenitors (Figure 8A) [29]. However, *Kras^G12D^* mice demonstrated consistent thymic enlargement compared to age-matched littermate controls, even prior to the onset of T-ALL (Figure 8B). Immunophenotyping of primary thymocytes demonstrated an essentially normal distribution of CD4+ and CD8+ expression, with a slight trend toward an increased number of CD4/CD8 double negative (DN) cells (after excluding Mac1+ and Gr1+ infiltrating myeloid cells) (Figure 8B). By contrast, further examination of the DN compartment using the cell surface markers CD44 and CD25 uncovered skewed development in *Kras* mutant mice (Figure 8C). Together, these data demonstrate that oncogenic *Kras^G12D^* perturbs thymic homeostasis, particularly in early stages of thymocyte maturation.

## Discussion

We find that oncogenic *Kras^G12D^* expression in HSCs confers a strong in vivo growth advantage, increases proliferation, and results in MPD and T-ALL. In MPD, as in normal marrow, stem cell activity is restricted to the Flk2^−^ LSK population, which represents less than 0.1% of nucleated marrow cells. While pathologic behaviors of more mature cells may contribute to tissue infiltration, anemia, and organomegaly, self-renewal is confined to this very primitive population. Therefore, hyperactive Ras signaling promotes excess proliferation in multiple hematopoietic compartments without immortalizing non–self-renewing cells. Similar data have been described in murine models of MPD based on *BCR-ABL* overexpression or loss of *JunB*, both of which also deregulate cytoplasmic signaling networks [5,30]. By contrast, recent experiments have provided direct evidence that some oncogenic transcription factors allow committed myeloid progenitors to acquire self-renewal ability [3,4]. Taken together, studies of myeloid oncogenes performed to date support the general idea that mutations that predominately alter cytoplasmic signaling networks and those that affect transcription factors controlling cell fate decisions comprise discrete complementation groups for the fully transformed phenotype [31,32].

Our limit dilution transplantation data demonstrate that oncogenic *Kras* confers a dramatic growth advantage in the HSC compartment. Under stringent conditions in which the contribution of a single WT HSC can barely be detected, the progeny of one (or at most three) *Kras^G12D^* HSC comprise a substantial fraction of the hematopoietic compartment. These studies provide direct experimental evidence that the outgrowth of malignant cells in MPD can be attributed to hyperactive Ras signaling in HSC. Our data suggest a pathogenic model in which JMML or CMML is initiated by a somatic mutation that deregulates Ras signaling in a single HSC. This idea is consistent with limited data from human patients and xenograft studies that implicate the HSC as the cell of origin for JMML [33–38].

To begin to address the mechanism by which mutant HSCs outgrow their WT counterparts, we analyzed the cell cycle in *Kras^G12D^* Flk2^−^ LSK cells and found they are preferentially in cycle. The overexpression of cyclin D1 in *Kras^G12D^* cells we observed is consistent with many prior studies in cultured cell lines engineered to overexpress oncogenic Ras [39,40]. Intriguingly, HSCs in mice lacking D-type cyclins demonstrate severe proliferative defects and accumulate in the G_0_ and/or G_1_ phases of the cell cycle [41]. If increased cyclin D1 levels conversely result in excessive proliferation of HSC, then MPD in *Kras^G12D^* mice may be mediated in part by cyclin D1, similar to the requirement for cyclin D1 in a model of Ras-mediated breast cancer [42].

We demonstrate a substantial early growth advantage of HSCs that express *Kras^G12D^*; however, it is also possible that oncogenic Ras expression has a negative long-term impact on HSC function. Increased proliferation or oncogenic stress may ultimately detract from self-renewal capacity. Reduced HSC fitness was observed in *Pten^−/−^* mice, in which phosphatidylinositol 3-kinase signaling is hyperactive [43,44]. Similar effects of *Kras^G12D^* are suggested by the reduced numbers of HSCs in *Kras^G12D^* mice, although this finding could also reflect changes in the composition of the Flk2^−^ LSK population or cell-extrinsic effects related to alteration of the marrow microenvironment. The rapid demise of primary *Kras^G12D^* mice from MPD, and of transplant recipients from T-ALL, precluded serial transplantation experiments to test the long term fitness of *Kras^G12D^* HSC. In addition, the 5-fold discrepancy between HSC numbers that were measured by flow cytometry versus limit dilution transplantation suggests a defect in engraftment of *Kras^G12D^* HSC. This idea is consistent with a prior report in which retroviral transduction of mutant *NRAS* appeared to reduce engraftment potential [45], and with extensive data showing that proliferating HSCs fail to engraft efficiently [46–50].

Despite the reduced number of HSC in the bone marrow of *Kras^G12D^* mice, our data are not entirely consistent with the idea that *Kras^G12D^* is a cell-intrinsic negative regulator of HSC self-renewal. If it were, we would expect specific loss of *Kras^G12D^* cells and outgrowth of WT or *Kras^LSL-G12D^* cells, because conditional models using *Mx1-Cre* typically retain a small pool of cells with the unrearranged locus [51,52]. However, we observed preferential retention of *Kras^G12D^* cells, even within the diminishing Flk2^−^ LSK compartment. This observation suggests that residual WT HSCs are unable to compensate for the reduced HSC number. Therefore, we favor the hypothesis that the reduction in HSC number in *Kras^G12D^* mice reflects a disordered bone marrow microenvironment with reduced supportive capacity rather than a purely cell-intrinsic effect of *Kras^G12D^* in HSC.

The natural history of hematologic disease was different in primary *Kras^G12D^* mice than in recipients of transplanted *Kras^G12D^* HSC. A subtle but important finding is that MPD is established earlier in primary *Kras^G12D^* mice than in transplanted recipients. (Figure 5 and [13]; also see [16,53]). There are several possible explanations for this observation. The hematopoietic microenvironment in young mice may be more permissive for MPD than the irradiated bone marrow of an adult. Additionally, pIpC administration in *Mx1-Cre* mice may quickly create a field of *Kras^G12D^* myeloid progenitors that contributes to the rapid evolution of MPD in primary mice through cytokine-mediated autocrine and/or paracrine mechanisms [54]. It is also possible that nonhematopoietic stromal cells in *Mx1-Cre, Kras^LSL-G12D^* mice express K-Ras^G12D^ and contribute to the rapid onset of MPD in primary *Kras^G12D^* mice.

The kinetics of MPD development relate directly to the high frequency of T-ALL we observed in transplanted recipients as compared to primary *Kras^G12D^* mice. The apparent incidence of T-ALL is highly subject to selection bias, because animals that die from MPD cannot be evaluated for subsequent emergence of T-ALL. For example, we have observed ∼10%–15% of *Kras^G12D^* mice develop T-ALL on an inbred C57BL/6 strain background (unpublished data), but median time to death from MPD is shorter than the typical latency of T-ALL, and lymphoid tumors exclusively appear in mice with a relatively late onset of MPD. Similarly, we have not observed spontaneous T-ALL in F1 (C57BL/6 × 129Sv/Jae) mice, which die from MPD at a younger age than the C57BL/6 strain described here ([13] and unpublished data). In transplant recipients, aggressive T-ALL arose in mice that also had evidence of underlying MPD that was not yet severe enough to kill the animal. Together, these observations suggest that the attenuation of MPD in transplant recipients was central to the apparent increase in the incidence of T-ALL in the transplant setting.

Cooperation of hyperactive Ras and deregulated Notch signaling in T-ALL has recently been shown [55–57]. Our data extend these studies by demonstrating the remarkable efficiency with which *Kras^G12D^* HSCs can initiate T-ALL, and delineating how multiple cell types may participate in the stepwise acquisition of oncogenic mutations in hematologic cancers. In the *Mx1-Cre, Kras^LSL-G12D^* model, T-ALL is initiated by oncogenic *Kras* expression in HSC, but full transformation occurs when cooperating *Notch1* mutations arise in a T-lineage cell. In this sense, both *Kras^G12D^* HSCs and the fully transformed thymocytes can be considered different types of malignant stem cells with distinct leukemogenic potentials.

One potential implication of these results is that the initiating *Kras* mutation creates conditions favorable for acquisition of cooperating mutations by increasing the size of susceptible lymphoid progenitor pools and/or conferring resistance to apoptotic signals during thymic selection. *Kras^G12D^* appears to most greatly affect the DN population that is characteristically undergoing TCR rearrangement, selection, and proliferation [58,59]. Interestingly, K-Ras^G12D^ protein expression may substitute for the pre-T cell receptor rearrangement at this critical checkpoint, thereby allowing propagation of thymocytes that would normally be edited [60]. Consistent with this idea, a patient with impaired lymphoid homeostasis and multiple lymphoid malignancies was recently reported to have a germline *NRAS^G13D^* mutation, and oncogenic *NRAS* suppressed apoptosis of lymphocytes after cytokine withdrawal [61]. By contrast, Kindler et al. recently reported reduced thymic cellularity in *Mx1-Cre, Kras^G12D^* mice [56]. We speculate that the proliferative effects of K-Ras^G12D^ in the T cell compartment were obscured in those studies by the short interval between pIpC injection, which induces systemic interferon production, and histologic analysis.

The idea that patients may harbor a variety of genetically distinct LSC is consistent with studies of patients with chronic myeloid leukemia in blast transformation [62], and has important therapeutic implications. The need to eliminate partially transformed but self-renewing cells, like *Kras^G12D^* HSCs, will depend on their propensity to initiate a life-threatening disease. Targeted therapies that are directed against onco-proteins such as K-Ras^G12D^ will effectively eliminate premalignant clones only if the targeted lesions are initiating rather than secondary mutations. For example, inhibition of Notch signaling is an attractive therapeutic strategy for T-ALL that is being investigated in the clinic. However, if these cancers arise from aberrant HSCs that do not contain a *NOTCH1* mutation and are not eradicated by treatment, relapse could occur through the acquisition of distinct cooperating mutations in a self-renewing preleukemic population. Consistent with this idea, studies of human T-ALL suggest that *NOTCH1* mutation occurs as a secondary mutation in at least some cases, with some patients developing recurrent disease having distinct *NOTCH1* alleles [63].

Finally, our data have implications for understanding the nature of cancer stem cell populations in nonhematopoietic malignancies. *KRAS* is the most frequent target of dominant oncogenic mutations in human cancer, and it is particularly important in carcinomas of the lung, pancreas, and colon. Analogous cancers arise in strains of mice expressing conditional oncogenic *Kras* alleles in these tissues [64–68]. Importantly, whereas oncogenic *Kras* expression efficiently initiates tumorigenesis in murine lung and pancreas, colon cancer is observed only when the tumor suppressor *Apc* is inactivated as well [68]. These data are consistent with studies of human patients, which imply that *KRAS* mutation occurs early in pancreatic cancer but typically after *APC* mutation in colon carcinoma [69–71]. Lung cancer in *Kras^G12D^* mice appears to be initiated in a distinct bronchio-alveolar stem cell population [72]. On the basis of these observations and our data, we speculate that, like HSCs, cells initiating pancreatic and lung cancer will possess inherent self-renewal potential, and that *KRAS* mutations only contribute to colon tumorigenesis in cells that have already acquired a mutation that enhances self-renewal. Uncovering specific proteins and pathways that are essential for the self-renewal and survival of *Kras* mutant cancer stem cell populations may reveal novel targets for therapeutic intervention in a variety of human cancers.

## Materials and Methods

### Ethics statement.

All animals were handled in strict accordance with good animal practice as defined by the relevant national and local animal welfare bodies, and all animal work was approved by the institutional animal research committee (University of California, San Francisco IACUC).

### Mouse strains.

Animals were housed in a barrier facility at the University of California, San Francisco. All mice were of the C57BL/6 strain. As described [13], 21-d-old *Mx1-Cre, Kras^LSL-G12D^* were injected IP with 250 μg pIpC (Sigma). *ROSA26 LSL-YFP* reporter mice were a gift from C. Lowell. *Ptprc* (CD45) congenic mice were from Jackson Labs.

### Colony assays.

To assay CFU-GM, nucleated bone marrow cells (10^5^) or splenocytes (2 × 10^5^) were suspended in 1 ml methylcellulose medium (M3231, StemCell Technologies) with 10 ng/ml murine GM-CSF (PeproTech). Colonies were counted after 8 d. To assay Flk2^−^ LSK cells, 100 sorted cells were plated in 1 ml methylcellulose medium (M3434, StemCell Technologies), which is provided containing IL-3, IL-6, stem cell factor, and erythropoietin, and incubated at 37 °C for 14 d. For *Kras* genotyping, individual colonies were picked with a 10 μl pipette and frozen overnight in 10 μl ddH_2_O prior to PCR analysis [13].

### Flow cytometry.

Flow cytometry was performed as described [21,24]. Staining was carried out in FACS Staining Buffer (FSB; HBSS with 2% heat inactivated FCS) at 0 °C unless otherwise stated. HSCs were defined as Lin^−/lo^ Sca1^+^ c-kit^+^ Flk2^−^, and CMP as Lin^−/lo^ Sca1^−^ c-kit^+^ CD34^+^ FcγR^−^. Antibodies were from eBioscience except as specified. To identify HSCs, cells were first stained for 1 h with unconjugated lineage antibodies: CD3 (17A2, BioLegend); CD4 (RM4–5); CD5 (53–7.3); CD8 (53–6.7); B220 (RA3-6B2); Ter119 (TER-119); Mac1 (M1/70); Gr1 (RB6-8C5). Cells were washed then incubated for 30 min with TriColor goat-anti-rat F(ab′)_2_ (Invitrogen) and murine IgG (Sigma). Cells were washed again, stained for 30 min with Pacific Blue anti-Sca1 (D7, BioLegend); APC anti-c-kit (2B8, BioLegend); PE anti-Flk2 (A2F10, BioLegend); and 7-AAD (Sigma) 5 μg/ml for dead cell exclusion. IL-7Rα was detected using a PE conjugate (eBioscience). In this case, or when analyzing YFP+ cells, Flk2 was detected with biotinylated anti-Flk2 and APC-Alexa Fluor 750 streptavidin (Invitrogen). We found no difference in Flk2^−^ LSK staining when Mac1^+^ cells were identified an on independent channel but not excluded. For myeloid progenitor analysis, PE anti-Flk2 was replaced with PE anti-FcγRII/III (93) and FITC anti-CD34 (RAM34), and cells were stained for an additional 30 min prior to analysis. Mature cells were assigned lineage using Pacific Blue anti-Mac1 and anti-Gr1 (myeloid), FITC anti-CD3 and anti-CD5 (T), and PE anti-B220 (B). T cell subsets in mice with T-ALL were distinguished with Pacific Orange anti-CD4 (Invitrogen), APC anti-CD8, FITC anti-CD3, and PE anti-CD5. To discern origin of cells in chimeric mice, antibodies to CD45.1 (A20; PE-Cy7) and CD45.2 (104; Alexa Fluor 700) were added. Primary thymocytes were analyzed with PE-Cy7 anti-CD4, Alexa 647 anti-CD8, PE anti-CD25, Pacific Blue anti-CD44, and FITC anti-Mac1 and anti-Gr1.

Prior to sorting, c-kit^+^ cells were enriched with anti-CD117 microbeads and an AutoMACS (Miltenyi). Cell sorting was performed on a FACSAria and flow cytometry on an LSRII, both using FACSDiva software (BD). Data were analyzed using FlowJo software (TreeStar).

### Transplantation.

Recipients received a single fraction of 950 rads for lethal irradiation, or 450 rads for sublethal irradiation, from a cesium source. Donor cells were prepared in 100 to 200 μl of FSB, and injected retro-orbitally into anesthetized mice. Recipients received water with neomycin and polymyxin for 2 wk. Blood counts were monitored monthly using a Hemavet 950FS (Drew Scientific).

Limit dilution was performed as described [73]. Lethally irradiated recipients were transplanted with 1 × 10^4^, 3 × 10^4^, or 1 × 10^5^ nucleated bone marrow cells along with 10^6^ unfractionated WT bone marrow cells for radioprotection. Peripheral blood was analyzed monthly by flow cytometry; mice with detectable engraftment in myeloid, B and T lineages 2 mo after transplantation were scored as positive and L-Calc software (StemCell Technologies) was used for statistical analysis.

For the CFU-S_8_ assay, lethally irradiated mice received 10^5^ CMPs without support cells. Spleen colonies were observed 8 d later by gross examination at harvest and after 48 h fixation in 10% formalin. Sections were stained with hematoxylin and eosin.

### *Notch1* analysis.

Cells were lysed in 1% NP-40 with 30 mM NaF, 30 mM β-glycerophosphate, 20 mM Na_4_P_2_O_7_, 1 mM Na_3_VO_4_, and Complete (Roche) and analyzed after SDS-PAGE using cleaved Notch1 and β-actin antibodies (Cell Signaling). Genomic DNA was PCR-amplified sequenced bidirectionally using 5′-ATAGCATGATGGGGCCACTA-3′ and 5′-GCCTCTGGAATGTGGGTGAT-3′.

### Cell cycle analysis.

Staining with 7-AAD and pyronin Y was performed as described [74] in nucleic acid staining solution (NASS; 0.1M phosphate-citrate buffer [pH 6.0] [Sigma], 5mM EDTA, 0.15M NaCl, 0.5% BSA), with 0.02% saponin (Sigma). Nucleated bone marrow cells were stained for 30 min with FITC lineage antibodies (CD3, CD4, CD5, CD8, B220, Ter119, and Gr1), Pacific Blue anti-Sca1, unconjugated anti-CD16/32 (2.4G2, UCSF hybridoma core), and biotinylated anti-Flk2. Cells were washed and then stained with APC-Alexa Fluor 750 streptavidin. Cells were washed again and resuspended in 500 μl of NASS with 1 μg/ml 7-AAD (Sigma), and incubated at room temperature for 30 min, then on ice for 5 min. Pyronin Y (Sigma) was then added to 1 μg/ml, and cells were incubated for an additional 10 min before being washed and resuspended in 200 μL of FSB. Cells were finally stained with APC anti-c-kit and FITC anti-Mac1. Differences in the quiescent fraction of HSCs were analyzed using an unpaired *t*-test.

### Quantitative real-time PCR.

The assay was performed as described [50]. Flk2^−^ KLS cells from animals pooled by genotype were double-sorted directly into RNA binding/lysis buffer from the RNEasy kit (Qiagen), and total RNA was extracted per instructions. First strand cDNA synthesis was performed using a SuperScript III kit (Invitrogen) per manufacturer's instructions. Reactions were performed in an ABI-7900 sequence detection system using SYBR green according to manufacturer's instructions (Applied Biosystems). Each amplification was performed in 10 μl with a template cDNA equivalent of 100 sorted HSCs. Each sample was tested in triplicate with each primer pair, and normalized to β-actin expression. Due to limiting numbers of doubly sorted cells, final cell purity was not analyzed; however the staining characteristics of c-kit-enriched and singly sorted cells are presented in Figure S1.

### Poisson statistics.

To analyze repopulation by a single HSC, we identified a cohort of mice receiving a cell dose yielding engraftment in only 50% of recipients. The Poisson distribution indicates that the probability of a mouse receiving *k* HSCs is given by

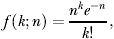
where *n* is the average HSC number per mouse. The average that yields *k* = 0 HSC at a rate of 0.5 is given by 0.5 = *e* ^−*n*^, which is solved to give *n* = 0.693. Using this value for the average number of HSC per mouse, the Poisson distribution can be used to estimate the likelihood of any mouse receiving a given number of HSC: *f*(0) = *e* ^−ln 2^ = 0.5; *f*(1) = (ln 2)*e* ^−ln 2^ = 0.346; *f*(2) = 1/2 (ln 2)^2^ *e* ^−ln 2^ = 0.120; *f*(3) = 1/6 (ln 2)^3^ *e* ^−ln 2^ = 0.028. The proportion of mice expected to receive four or more HSCs is the remainder, 1 − (0.5 + 0.346 + 0.12 + 0.028) = 0.006, or 0.6%.

## Supporting Information

Figure S1 Purity of Cells Sorted for RNA Purification Cells expressing c-kit were enriched by magnetic bead isolation, then doubly sorted by FACS. FACS data for the first and second sorts for typical examples of WT and *Kras^G12D^* mice are shown.
(740 KB TIF)

Figure S2 Flk2^−^ LSK Cell Number in 8-wk-Old *Kras^G12D^* Mice Flow cytometry was performed to enumerate Flk2^−^ LSK cells in bone marrow (*n =* 4; *p <* 0.05) and spleens (*n =* 6; *p <* 0.05) harvested from 8-wk-old *Kras^G12D^* or WT mice. Data were analyzed as in Figure 2. Combined numbers in marrow and spleen were not significantly different between genotypes (*p =* 0.27). Data are pooled from two independent experiments; error bars show standard error of the mean (SEM). Statistical significance was evaluated with the unpaired *t*-test.
(80 KB TIF)

Figure S3 *Kras^G12D^* HSCs Dominate the Stem Cell Compartment by 3 mo after Transplantation Mice transplanted with 500 CD45.1 *Kras^G12D^* and 500 CD45.1/CD45.2 WT HSCs were sacrificed (A) 2 mo (*n =* 6) or (B) 3 mo (*n =* 2) after transplantation, and their chimerism was assessed by flow cytometry. The percentage of graft cells derived from the *Kras^G12D^* HSCs is shown for Flk2^−^ LSK cells (black bars), CMPs (dark grey bars), granulocyte-macrophage progenitors (GMP, light grey bars), and mature (Mac1/Gr1+) circulating myeloid cells (open bars). GMPs were defined as Lin^−/lo^ Sca1^−^ c-kit^+^ FcγRII/III^+^. Animals lacking any graft-derived contribution to the HSC compartment have been omitted.
(168 KB TIF)

Table S1 Outcomes of Individual Limit Dilution Transplantation Experiments The indicated numbers of mice were transplanted with 10,000, 30,000, or 100,000 nucleated bone marrow cells, and the proportions with detectable multilineage engraftment were determined 2 mo later. The number of repopulating units was calculated using the totals from these four experiments with L-Calc software (StemCell Technologies).
(47 KB PDF)

## Abbreviations

7-AAD: 7-aminoactinomycin D
CFU-GM: granulocyte-macrophage colony forming unit
CFU-S8: day 8 spleen colony-forming unit
CMML: chronic myelomonocytic leukemia
CMP: common myeloid progenitor
DN: double negative
HSC: hematopoietic stem cell
JMML: juvenile myelomonocytic leukemia
LSC: leukemia stem cell
LSK: Lin−/lo Sca1+ c-kit+
LSL: loxP-STOP-loxP
MPD: myeloproliferative disorder
pIpC: polyinosinic-polycytidylic acid
SEM: standard error of the mean
T-ALL: T lineage acute lymphoblastic leukemia/lymphoma
WT: wild type
YFP: yellow fluorescent protein
