# Supplementary material for: Oncogenic Kras Initiates Leukemia in Hematopoietic Stem Cells
Source: PLoS Biol. 2009 Mar 17;7(3):e1000059. doi: 10.1371/journal.pbio.1000059 (PMC2656550; doi:10.1371/journal.pbio.1000059)
Supplement: Supplementary file 1 [file pbio.1000059.st001.doc]

|  |  | # Engrafted / #Transplanted | |
| --- | --- | --- | --- |
| Experiment | Dose NBMC | WT | *Kras^G12D^* |
| 1 | 10,000 | 2/3 | 0/3 |
|  |  |  |  |
| 2 | 10,000 | -- | 0/3 |
|  | 30,000 | -- | 0/2 |
|  | 100,000 | -- | 1/1 |
|  |  |  |  |
| 3 | 10,000 | 0/1 | 0/1 |
|  | 30,000 | 2/3 | 0/3 |
|  | 100,000 | 1/1 | 2/3 |
|  |  |  |  |
| 4 | 10,000 | -- | 0/2 |
|  | 30,000 | 2/2 | -- |
|  | 100,000 | 6/6 | 1/3 |
|  |  |  |  |
|  |  |  |  |
| Totals | 10,000 | 2/4 | 0/9 |
|  | 30,000 | 4/5 | 0/5 |
|  | 100,000 | 9/9 | 4/7 |

**Tabe S1.Outcomes of individual limit dilution transplantation experiments.**

The indicated numbers of mice were transplanted with 10,000, 30,000, or 100,000 nucleated bone marrow cells, and the proportions with detectable multilineage engraftment were determined 2 mo later. The number of repopulating units was calculated using the totals from these four experiments with L-Calc software (StemCell Technologies).
